# Defining the Environmental Adaptations of Genus *Devosia:* Insights into its Expansive Short Peptide Transport System and Positively Selected Genes

**DOI:** 10.1038/s41598-020-58163-8

**Published:** 2020-01-24

**Authors:** Chandni Talwar, Shekhar Nagar, Roshan Kumar, Joy Scaria, Rup Lal, Ram Krishan Negi

**Affiliations:** 1 Department of Zoology, University of Delhi, Delhi, 110007 India; 2 P.G. Department of Zoology, Magadh University, Bodh-Gaya, 824234 Bihar India; 3 Department of Veterinary and Biomedical Sciences, South Dakota State University, Brookings, SD USA; 4 South Dakota Center for Biologics Research and Commercialization, Brookings, SD USA; 5 NASI Senior Scientist Platinum Jubilee Fellow, The Energy and Resources Institute, Darbari Seth Block, IHC Complex, Lodhi Road, New Delhi 110003 India

**Keywords:** Microbiology, Genome informatics

## Abstract

*Devosia* are well known for their dominance in soil habitats contaminated with various toxins and are best characterized for their bioremediation potential. In this study, we compared the genomes of 27 strains of *Devosia* with aim to understand their metabolic abilities. The analysis revealed their adaptive gene repertoire which was bared from 52% unique pan-gene content. A striking feature of all genomes was the abundance of oligo- and di-peptide permeases (oppABCDF and dppABCDF) with each genome harboring an average of 60.7 ± 19.1 and 36.5 ± 10.6 operon associated genes respectively. Apart from their primary role in nutrition, these permeases may help *Devosia* to sense environmental signals and in chemotaxis at stressed habitats. Through sequence similarity network analyses, we identified 29 Opp and 19 Dpp sequences that shared very little homology with any other sequence suggesting an expansive short peptidic transport system within *Devosia*. The substrate determining components of these permeases *viz*. OppA and DppA further displayed a large diversity that separated into 12 and 9 homologous clusters respectively in addition to large number of isolated nodes. We also dissected the genome scale positive evolution and found genes associated with growth (exopolyphosphatase, HesB_IscA_SufA family protein), detoxification (*moeB, nifU*-like domain protein, alpha/beta hydrolase), chemotaxis (*cheB, luxR*) and stress response (*phoQ, uspA, luxR, sufE*) were positively selected. The study highlights the genomic plasticity of the *Devosia* spp. for conferring adaptation, bioremediation and the potential to utilize a wide range of substrates. The widespread toxin-antitoxin loci and ‘open’ state of the pangenome provided evidence of plastic genomes and a much larger genetic repertoire of the genus which is yet uncovered.

## Introduction

*Devosia* comprises a group of motile, gram-negative bacteria within the class *Alphaproteobacteria* and family *Hyphomicrobiaceae*^1^. The first recognized species of the genus was *Pseudomonas riboflavina* IFO13584 described by Foster in 1944^2^ from riboflavin-rich soil which was reclassified into *Devosia riboflavina* in 1996^1^. Since then, many members of this genus have been reported from diverse ecological niches. Although their distribution is ubiquitous including their presence in human cerebrospinal fluid^3^, nodules of legume plants^4,5^ and beach sediment^6^, members of this genus have been mainly reported from soils contaminated with hexachlorocyclohexane (HCH)^7,8^, mycotoxins (deoxynivalenol)^9,10^ and other hydrocarbon pesticides^11^.

In an effort to characterize the culturable diversity of soil contaminated with HCH^12–18^, we isolated and characterized four novel members of the genus *Devosia viz. D. chinhatensis* IPL18^7^, *D. crocina* IPL20^8^, *D. albogilva* IPL15^8^ and *D. lucknowensis* L15^19^. Although isolated from HCH contaminated soil, these isolates were not able to degrade HCH isomers^8^. However, members of the genus are best studied for their potential to degrade several toxic compounds, establishing their promising candidature for bioremediation^2,9^. Previous studies have aimed to characterize their metabolic routes of detoxification^20^. In spite of their abundance in culture collections and public repositories, the genetic repertoire that enables them to survive in harsh environments have not been elucidated. Here, we report the first comparative genomic study of 27 members of genus *Devosia*, which provides valuable insights into their adaptations, the role of environment in shaping their genomes and the degree of genomic evolution in response to different environmental pressures.

Our study suggested the influx of new metabolic capabilities into the “open” pangenome of *Devosia*. Besides, the phylogenetic relationships of the group were fairly consistent. The study revealed that the genomes harbor a large diversity of transporters involved in uptake of di- and oligo-peptides from the environment. These peptide transport systems enable bacteria to take up short peptides of different amino-acid composition for satisfying nutritional demands and have been extensively studied in species of *Lactococcus* and *Staphylococcus*^21,22^. Besides increasing nutritional fitness, these permeases are also shown to be involved in signaling and virulence in *Staphylococcus aureus, Borrelia burgdorferi* and *Bacillus thuringiensis*^23–26^. Here, our analysis revealed the high diversity of these permeases encoded within genus *Devosia* for enabling efficient nutrient utilization and cell signaling required at such environments. Additionally, the large diversity of their substrate binding components reflected their wide range of substrates utilization. Positive evolution and selection of genes associated with growth and utilization of toxins highlights future applications in bioremediation. Further, the genomic repertoire adapted for utilization of organic sulfur, phosphorus and aromatic compounds are presumed to enable the members of the genus *Devosia* to survive in harsh sites. The presence of toxin-antitoxin (TA) loci within their genomes provided evidence of enhanced genome plasticity for maintaining a wide range of biological functions including stress response.

## Results and Discussion

### Genomic features

Genome analysis of twenty seven strains of the genus *Devosia* showed >96% completeness establishing the reliability of the datasets for comparative analyses. The overall genomic features of the strains are listed in Table 1. The genome size ranged from 3.5 to 5.8 Mbp with an average genome size of 4.3 ± 0.6 Mbp. Notably, the strains isolated from HCH contaminated sites namely, IPL-18, L15 and IPL-20 represented the three smallest genomes. It is difficult to explain the minimum genomic size of the organisms at such contaminated and nutrient depleted sites. However, in a previous study, where we isolated and described a *Pseudomonas* species that has the smallest genome with respect to its neighbours, this was attributed to the HCH isomer pressure shaping the genomic repertoire^27^. IPL18 and L15 also lacked genetic potential for utilization of organic phosphorus, rather found in other genomes. It is likely that the organisms lost several accessory gene clusters as part of adaptations to survive at HCH rich habitat. The two largest genomes of Root105 and Root413D1 harboured several hypothetical proteins in singletons along with the genes involved in drug resistance (daunorubicin and doxorubicin), serralysin and leukotoxin, type I secretion system, adhesion protein BmaC and polyamine synthesis proteins. These proteins are associated with protection, adhesion and biofilm formation and may facilitate the colonization of these strains in plant roots^28,29^. The %GC contents varied between 60.5–65.9% with an average of 63 ± 1.7%. Each genome, on an average consisted of 4,330 ± 620.4 protein coding genes. The number of predicted coding sequences correlated positively with the genome size (PMCC, r = 0.99). The large difference with respect to genome size and the coding potential among the species reflected towards the cadences in the genomic repertoire of the *Devosia* ecotypes in response to the different niches.

**Table 1:** General attributes of the *Devosia* genomes analyzed in this study.

Strain	Genome Size (bp)	No. of Contigs	GC Content (%)	CDS	rRNAs (5S, 16S, 23S)	tRNAs	CRISPRs	Source of Isolation	Accession Number	Reference
*Devosia insulae* DS-56	5,750,119	410	65.3	5632	1,1,1	50	—	Soil sample South Korea: Dokdo Island, East Sea of Korea	NZ_LAJE00000000.2	^98^
*D. limi* DSM17137	4,297,227	25	62.7	4183	2,1,1	48	—	Nitrifying inoculum of activated sludge in Gent, Belgium	NZ_FQVC00000000.1	^98^
*D. soli* GH2-10	4,136,371	48	61	4183	3,1,1	48	—	Greenhouse soil used to cultivate lettuce in Daejeon City, Korea	NZ_LAJG00000000.1	^98^
*D. epidermidihirudinis* E84	3,859,784	47	61.1	3745	2,2,2	49	—	Skin of medical leech *Hirudo verbana*, from Biebertal, Germany	NZ_LANJ00000000.1	Unpublished data
*D. riboflavina* IFO13584	5,052,234	113	61.8	5042	1,1,1	52	—	Riboflavin rich soil in Rahway, New Jersey	NZ_JQGC00000000.1	^99^
*D. chinhatensis* IPL-18	3,497,719	98	62.3	3437	2,2,2	48	—	Soil samples from an India Pesticide Limited plant at hexachlorocyclohexane (HCH) dump site, Lucknow, India.	NZ_JZEY00000000.1	^91^
*D. geojensis* BD-c194	4,465,063	207	65.9	4432	1,1,1	49	—	Diesel-contaminated soil in Geoje, Korea	NZ_JZEX00000000.1	^100^
*D. crocina* IPL-20	3,723,990	7	61.3	3706	1,1,1	45	1	Hexachlorocyclohexane (HCH)-contaminated site in Chinhat, Lucknow, India	NZ_FPCK00000000.1	This study
*D. psychrophila* CGMCC 1.10210	4,328,275	85	61.2	4353	1,1,1	49	—	Alpine glacier cryoconite, Tyrol, Austria	FOMB00000000.1	Unpublished data
*D. enhydra* ATCC 23634	4,220,684	5	65.6	4107	2,1,2	48	1	Freshwater from the Putah Creek overflow in Davis, Calif, California	NZ_FPKU00000000.1	Unpublished data
*D. lucknowensis* L15	3,719,665	3	62.9	3722	1,1,1	46	1	HCH contaminated pond soil in Ummari village, Lucknow, India	NZ_FXWK00000000.1	This study
*D. subaequoris* HST3-14	4,123,118	20	60.9	4165	3,1,1	48	—	Sediment sample from Hwasun Beach in Jeju, Republic of Korea	IMG Genome ID 2654587640	Unpublished data
*Devosia* sp. LC5	4,202,858	47	62.3	4217	2,2,2	48	—	Limestone Capitan Formation at −347 m in Lechuguilla Cave, New Mexico, U.S.A.	JNNO00000000.1	^101^
*Devosia* sp. H5989	4,594,249	1	64.8	4574	2,2,2	51	—	Human cerebrospinal fluid	NZ_CP011300.1	^3^
*Devosia* sp. Root436	3,919,001	16	63.8	3890	1,1,1	46	1	Root of *Arabidopsis thaliana* cultivated in greenhouse in Germany;Cologne	LMEM00000000.1	^102^
*Devosia* sp. Root685	4,397,456	5	61.5	4228	1,1,1	48	—	Root of *Arabidopsis thaliana* cultivated in greenhouse in Germany;Cologne	LMHK00000000.1	^102^
*Devosia* sp. A16	5,032,994	1	65.8	4992	2,2,2	57	—	Wheat field, China; Nanjing	NZ_CP012945.1	^10^
*Devosia* sp. 17-2-E-8	4,684,238	124	64	4584	2,1,1	49	—	Alfalfa soil sample that was enriched with *F. graminearum*-infested moldy corn for 6weeks, Canada;Ontario	JQGB00000000.1	^99^
*Devosia* sp. Root105	5,850,117	21	65.4	5737	1,1,1	51	—	Root of *Arabidopsis thaliana* cultivated in greenhouse in Germany;Cologne	LMCR00000000.1	^102^
*Devosia* sp. Root413D1	5,851,361	14	65.4	5716	1,1,1	50	—	Root of *Arabidopsis thaliana* cultivated in greenhouse in Germany;Cologne	LMEA00000000.1	^102^
*Devosia* sp. Root635	3,816,628	24	64.1	3748	1,1,1	48	1	Root of *Arabidopsis thaliana* cultivated in greenhouse in Germany;Cologne	LMGZ00000000.1	^102^
*Devosia nanyangense* DDB001	4,669,456	95	64	4578	1,1,1	49	—	Mycotoxin contaminated Wheat field soil in Nanyang, China	CCAO000000000.1	^9^
*Devosia* sp. S37	3,878,148	151	64.1	3878	1,1,1	55	—	Oil palm rhizospheric soil, Temerloh, Pahang, Malaysia	LVVY00000000.1	Unpublished data
*Devosia* sp. Leaf64	4,244,488	24	60.5	4206	1,1,1	48	—	*Arabidopsis thaliana* leaf natural site, Switzerland; Zurich	LMLO00000000.1	^102^
*Devosia* sp. Leaf420	4,219,583	16	60.7	4128	1,1,1	50	—	*Arabidopsis thaliana* leaf natural site, Switzerland; Zurich	LMQU00000000.1	^102^
*Devosia* sp. YR412	3,831,215	11	62.5	3755	2,2,2	51	—	*Populus* root and rhizosphere microbial communities from Tennessee, USA	FOFL00000000.1	Unpublished data
*Devosia* sp. I507	4,005,916	1	61.9	4021	2,2,2	48	—	Pit mud, Indian ocean	NZ_CP026747.1	Unpublished data

### Phylogenomics analyses

**Figure 1:**
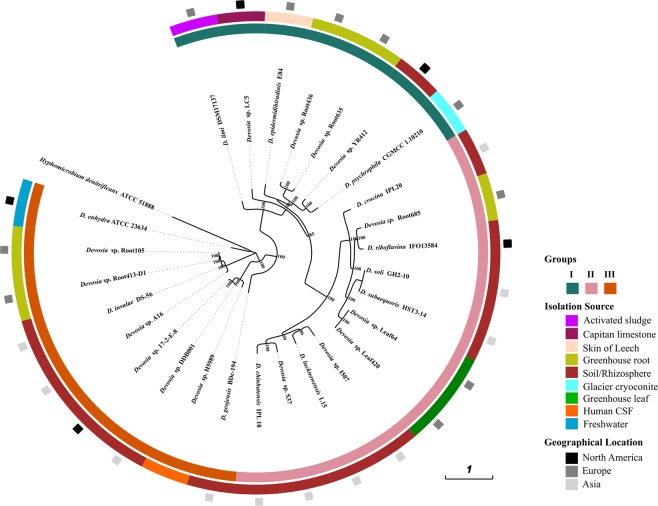
Phylogenomics analysis. The tree is based on the 400 conserved bacterial marker gene sequences constructed using maximum likelihood method with 1000 bootstrap replications. The innermost ring represents the three major groups of strains thus formed which are denoted as Group I, II and III. The colors in the middle ring represent the habitat of each strain and the outermost ring represents their geographic origin. The tree was constructed using iTOL (https://itol.embl.de/)^84^.

**Figure 2:**
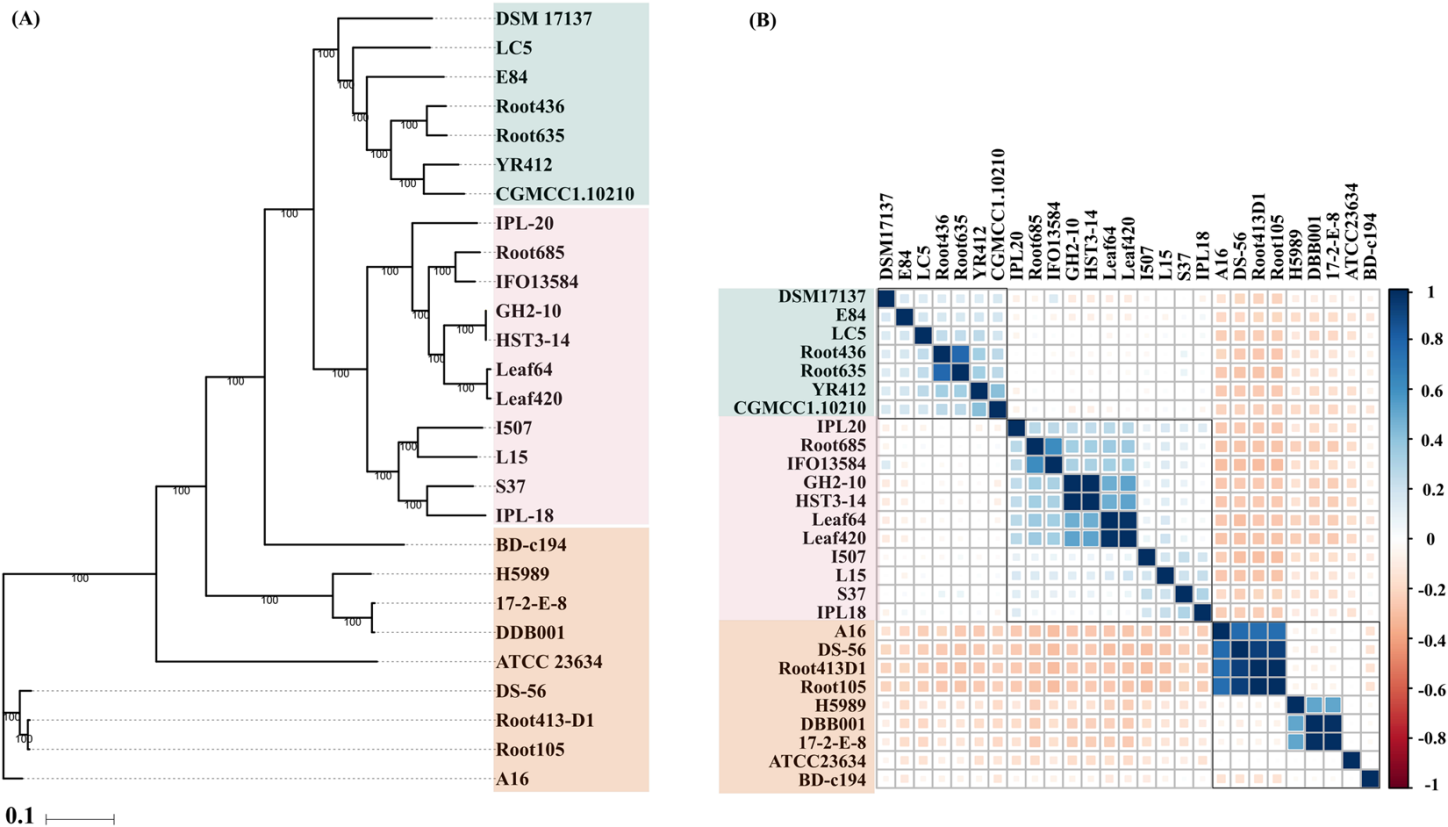
Phylogenomics analyses. (**A**) Maximum likelihood tree based on the single copy core genetic content of the 27 analyzed members of the genus *Devosia*. Bootstrap values calculated from 100 bootstrap repetitions are denoted. (**B**) Correlation between the genomes on the basis of blast based average nucleotide identity (ANIb) values. The blue and pink squares denote high and low correlation values for a pair of genomes and the corresponding values of predicted Pearson correlation coefficients (-1 to 1.0) are shown in the adjacent bar.

We deciphered the phylogenetic relationships of *Devosia* strains using marker genes, core genome and whole genome based average nucleotide identities. Maximum likelihood phylogeny based on the conserved set of 400 bacterial marker genes (Fig. 1)^30^ was reasonably consistent with that obtained from the concatenated alignments of 1,165 orthologous single copy core genes identified using OMCL algorithm (Fig. 2A). The phylogeny reconstructed from the whole genome wide ANIb also revealed identical topology (Fig. 2B). All the methods clearly resolved the genus into three different groups denoted as Group I, II and III with subclades (Figs. 1 and 2). Intriguingly, isolates from unrelated environments, for instance, CGMCC 1.10210 isolated from glacier cryoconite and YR412 isolated from rhizosphere clustered together while those from same habitats, such as isolates from *Arabidopsis* root appeared distantly in the phylogeny. This suggests that the role of environment in shaping bacterial genomes is still undefined.

We noticed high ANI values shared between the type strains *D. soli* GH2-10 and *D. subaequoris* HST3-14 (99.99%) with high percentage of conserved proteins (98.15%). However, as the percentage similarity shared between their submitted 16S rRNA gene sequences is less than 98.65%, it highlighted the need to redefine the boundaries for species demarcation due to low phylogenetic resolution of 16S rRNA marker gene^31^. Both the genomes were predicted to be 98.1% complete supporting the ANI based prediction. Similarly, we detected other pairs that might represent single species based on ANI values with the cutoff score of >95% defined for species demarcation that included the two DON degrading strains DDB001 and 17-2-E-8 (99%), *Arabidopsis* leaf isolates, Leaf64 and Leaf420 (96%), *Arabidopsis* root isolates, Root105 and Root413-D1 (98%). Moreover, the pairs also clustered together based on the comparative functional analysis while harboring the similar genetic repertoire. Further, the analysis showed that *D. soli* GH2-10 and *D. subaequoris* HST3-14 are likely the same species with a high ANIb value of 99.9% (Fig. 2B).

### Pangenome analysis

**Figure 3:**
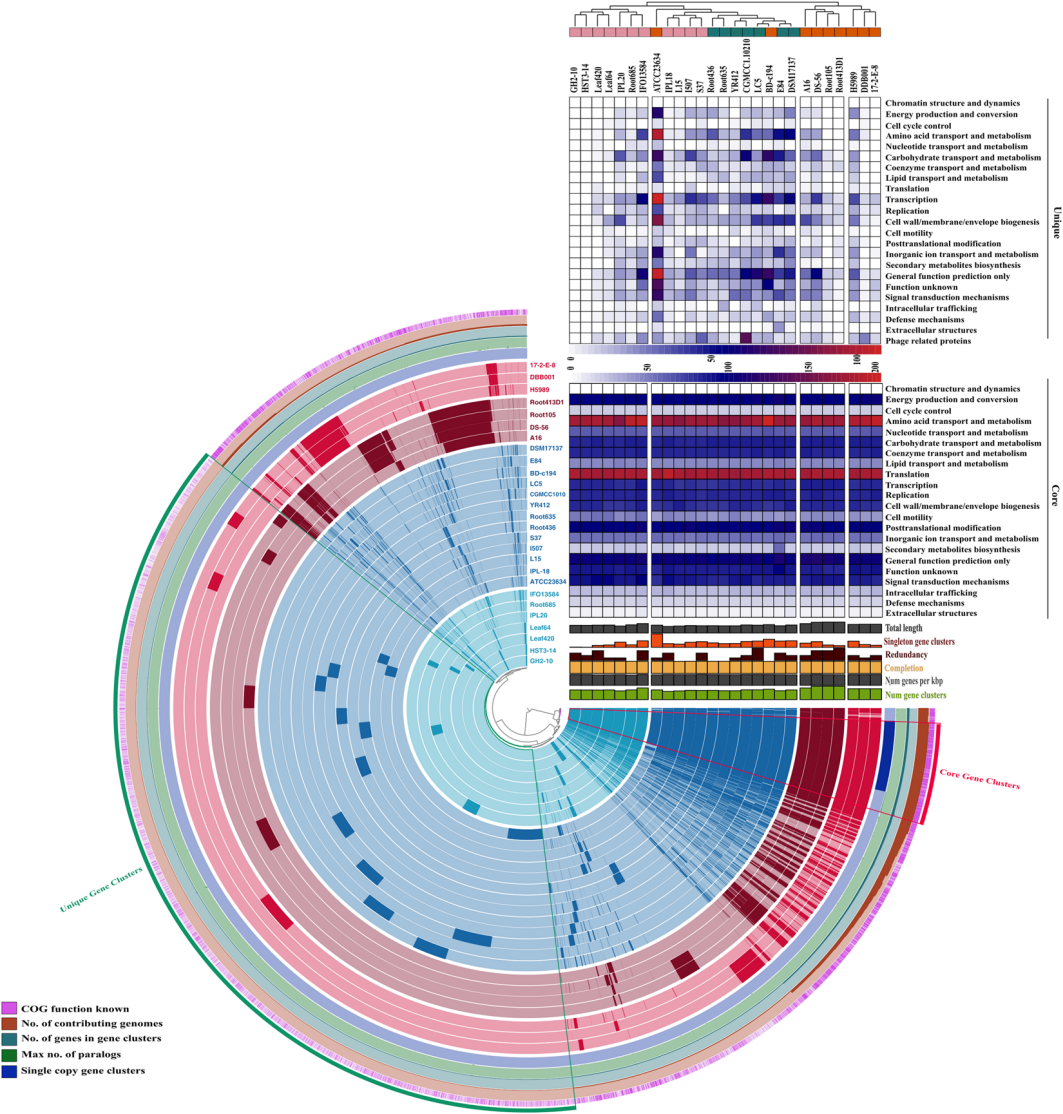
Pangenome analysis. Clustering of genomes based on the presence/absence patterns of 23,421 pangenomic clusters. The genomes are organized in radial layers as core, unique and accessory gene clusters [Euclidean distance; Ward linkage] which are defined by the gene tree in the center. The clades are colored based on the shared gene clusters as shown in the tree in the right top above the heatmaps and the phylogenomic groups of the strains are denoted by the corresponding colors in the pangenome tree as in Fig. 1. Heat maps denote the functions enriched in the core- (below) and strain-specific (top) gene contents based on annotated clusters of orthologous groups (COG) categories. The core- and strain-specific gene clusters are highlighted to distinguish them from dispensable genome. The figure was constructed using Anvi’o pangenomics workflow (http://merenlab.org/software/anvio/)^33^.

The pangenome of *Devosia* was analysed with aim to determine its genetic potential. The pangenome is defined as entire set of gene clusters present in a group and is constituted by the core and accessory genomes^32^. The core genome is formed of the conserved set of genomic functions found in all strains of the group. While accessory genome consists of the dispensable component which is present in a subset of genomes and the strain-specific content (singletons) that is unique to only one strain out of all the analysed genomes. The pangenome of *Devosia* was shown to be formed by 23,421 gene clusters (Distance: Euclidean; Linkage: Ward)^33^ that included 1,257 core, 10,383 dispensable and 11,781 strain-specific gene clusters. The small sized core (5.4%) and unique content (50.3%) forming more than half of the pangenome indicated that the species are highly diverged (Fig. 3). A robust analysis of the changes in pan- and core genome sizes upon sequential addition of genomes and their regression trends plotted as Tettelin best fit curve revealed indefinite increase in pangenome size up to the addition of the last genome. Therefore, the pangenome of *Devosia* may be classified as ‘open’ for expansion (Supplementary Fig. S1).

The distribution of strains based on the pangenomic clusters was deviated from the phylogenomic clustering which was partly reflected in the differences in the accessory genomic contents of Group III strains (Fig. 3). The core and unique gene clusters were further annotated into COG classes. The core genome was mostly conserved in the following: amino acid transport and metabolism (11.12%), translation and ribosomal biogenesis (11.27%), post translational modifications (6.42%), energy production and conversion (6.36%) and signal transduction (5.65%). As largely the species are soil microbes that inhabit intoxicated environments, their genomes are thus enriched in genes for efficient uptake of the restrained nutrients, sense chemotactic stimuli and transduce the signals for colonization. The classes for carbohydrate metabolism (5.44%), transcription (5.25%), replication (5.32%) and cell envelope synthesis (5.18%) were moderately abundant while intracellular trafficking, secondary metabolite synthesis, defense mechanisms and extracellular structures were limited in the core (0.5–2%). About 70% of the average genome size of the genus was not conserved indicating a high degree of genomic diversity. Isolate ATCC23634 was found to harbor highest numbers of singletons which is expected as it is the only isolate from freshwater (Table 1). Besides, it also harbored a large CRISPR locus with 16 spacer sequences unveiling its adaptive immunity resulting from previous viral encounters.

### Comparative functional profiles

**Figure 4:**
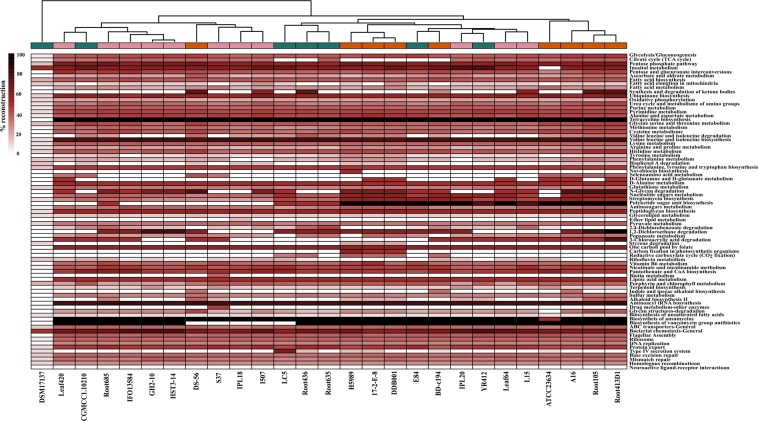
Comparative metabolic pathway analysis. The top metabolic pathways within each genome are compared based on their percentage reconstruction. A dendrogram constructed based on the metabolic profiles is shown at the top and the different phylogenetic groups are shown with corresponding colors. The heatmap was constructed using pheatmap^92^ in R (R Development Core Team, 2015).

To gain more insights into specific functions, the top metabolic pathways of the genus were minimally reconstructed within individual genomes. Interestingly, the phylogenetically consistent groups of strains displayed different functional profiles and revealed an altogether different clustering pattern (Fig. 4). It suggests that their functional profiles might have been selected by the environment owing to evolutionary processes such as gene gain or loss and lateral gene transfer. The top metabolic pathways that were reconstructed within the genomes involved metabolism of sugars, fatty acids and amino acids, biosynthesis of antibiotics such as tetracycline, ansamycins and vancomycins, flagellar assembly and chemotaxis, ABC class transporters and degradation of chlorinated hydrocarbon compounds. These abundant functions are anticipated to provide survival benefits to the strains at the diverse niches that they inhabit. Strain DSM17137 was uncovered to be the most diverged strain within the genus with respect to its overall functional profile as the top metabolic pathways could not be reconstructed within its genome (Fig. 4). A major difference in the clades thus obtained was observed in the genes for synthesis of polyketide sugars that are important antimicrobial agents^34^ indicating that defence is not a primary function and hence not a part of the core genome. Concurrently, we noted that the clustering based on functional profiles was not strictly habitat-dependent. For instance, the strains isolated from plant leaves, Leaf64 and Leaf420 showed key differences in selenoamino acids utilization and polyketide sugar unit biosynthesis. This may be explained based on the fact that the process of gene gain or loss does not necessarily occur at the same rate in the isolates from similar habitats and hence the differences were observed. Similarly, the isolates from HCH contaminated soils showed different profiles for degradation of 1,2-dichloroethane and 3-chloroacrylic acid and for synthesis and degradation of ketone bodies. This suggests their dynamic genome repertoire and that the strains might be in the process of acquiring the genes for degradation of chlorinated hydrocarbons at this site.

### Abundance of Oligo- and Di- peptide ABC transporters

**Figure 5:**
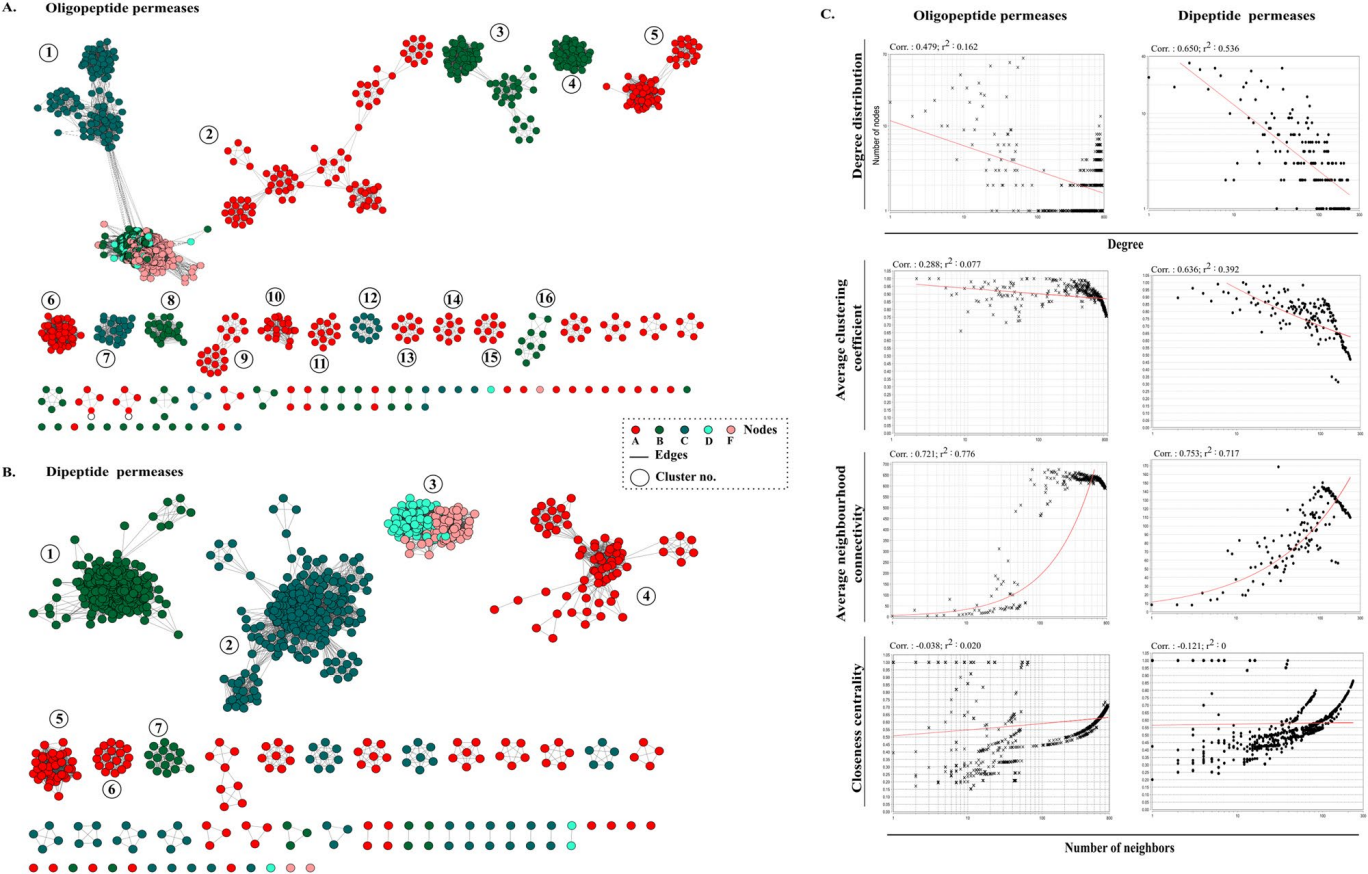
Sequence similarity network analyses. Diversity of (**A**) Oligopeptide (Opp) and (**B**) Dipeptide (Dpp) permeases in analysed genomes. The nodes represent sequences connected through edges if the similarity exceeds the cutoff score. The networks are thresholded at e-value cutoff of 1e-30 and 1e-25 respectively. The ABCDF components of the permeases are represented by different colors. The clusters are ranked in order of decreasing number of nodes. Clusters with more than 10 nodes are numbered. (**C**) Topological properties of the similarity networks: degree distribution, average clustering coefficient, average neighborhood connectivity and closeness centrality are plotted against the number of neighbors. The power law fit curves are shown within each graph.

As amino acid transport and metabolism emerged as one of the most abundant functions of the genus, we studied the genes of this class for determining the important survival strategies of *Devosia*. More precisely, we found these genomes to be enriched in the oligo-peptide permeases (Opp) and di-peptide permeases (Dpp). Opp and Dpp permeases are present in the bacterial membranes as multi-subunit protein complexes and function primarily in the uptake of peptides from the environment to serve as a source of carbon and nitrogen. These transport systems have been widely studied in species of *Lactococcus, Staphylococcus, Borrelia* and *Bacillus* where they have been shown to be involved in growth, signalling and virulence^21–26^. The permease complex has a typical structure of an ABC class transporter: a substrate binding protein OppA/DppA, two transmembrane proteins OppB/DppB and OppC/DppC and two membrane bound cytoplasmic ATP-binding proteins OppD/DppD and OppF/DppF^35^. The copy number of each of these transporters within the analysed strains is given in Supplementary Fig. S2. Their large diversity and abundance in *Devosia* was further checked by comparing these permeases with those in representative genomes (n = 27) of other genera of family *Hyphomicrobiaceae* (Supplementary Table S1). A large diversity in the organization of genes within operons was observed and many individual genes were found segregated throughout the genomes. As the presence of each of the gene in the cluster is not a prerequisite for the operon to be functional, their abundance might be an adaptation for uptake of large variety of peptides for optimal nutrition^36^. The gene copy number varied from 21 to as high as 93 Opp operon associated genes with an average of 60.7 ± 19.1 copies within each genome. Also, the genomes were abundant in Dpp permeases with 17 to 54 copies of associated genes within a genome and each genome carried an average number of 36.5 ± 10.6 genes. Their genetic diversity across the genus was determined by eliminating ~6.8% of the redundant sequences in each case (sequence identity = 100%) from a total of predicted 1,640 Opp and 986 Dpp sequences indicating high diversity of these transporters. An empirical measure of the diversity among the permeases and comparison of pairwise relationships was determined through sequence similarity network (SSN) analysis. In SSN, each protein sequence is represented by a node and any two nodes are connected by edges if they share more than the defined threshold similarity. The similarity networks for all non-redundant Opp and Dpp sequences were visualized, using the threshold pairwise Blastp e-value of 1e-30 and 1e-25 respectively. Each node in the resulting networks could not be connected with all other nodes through a finite path (Fig. 5A,B). OppABCDF and DppABCDF partitioned into 65 and 55 connected components respectively that included both homologous and heterologous clusters and isolated nodes. Through network analysis, we identified 29 Opp and 19 Dpp sequences that did not share homology with any other sequence suggesting an expansive short peptidic transport system within *Devosia*. Average neighborhood connectivity within the networks was interpreted as an increasing function in *k* both in case of Opp (correlation = 0.72, r^2^ = 0.77) and Dpp (correlation = 0.75, r^2^ = 0.71) suggesting scarce edges between low connected and highly connected nodes and highlighting the diversity among the sequences (Fig. 5C). Furthermore, closeness centrality that measures the closeness of a node with all other nodes was negatively correlated with the number of neighbors in both Opp (-0.038, r^2^ = 0.020) and Dpp (−0.121, r^2^ = 0) (Fig. 5C). More specifically, we analysed the diversity of substrate binding components (SBCs): OppA and DppA within these complexes. OppA partitioned into 12/29 isolated nodes while DppA constituted 9/19 isolated nodes. The network parameters are noted in Table 2. Notably, all the isolated nodes of SBCs belonged to the species of the Group III strains that were most diverged in phylogeny (Fig. 1). Both the networks were very sparse and analysis of the networks revealed that a random Opp sequence was similar to only 20.5% of all the sequences which was even less 6.2% in case of OppA (n = 343) (Table 2). At the same time, any random Dpp sequence was similar to only 5.5% of the sequences while the similarity between any two DppA sequences (n = 192) was estimated to be 8.3%. Phylogenetic diversity of these SBCs was further compared with those predicted in the representative genomes (n = 27) from other genera of family *Hyphomicrobiaceae* by constructing a neighbour joining tree (Supplementary Fig. S3).

**Table 2:** Parameters of the sequence similarity networks.

Network Parameters	Opp	OppA	Dpp	DppA
No. of Nodes	1529	343	919	192
No. of Edges	2,39,422	—	23,141	—
Average degree	313.17	21.24	50.36	15.94
Connected components	65	—	55	—
Isolated nodes	29	12	19	9
Network Density	0.20	0.06	0.05	0.08
Characteristic path length	1.92	—	2.02	—
Shortest path	38%	—	18%	—
Network centralization	0.298	0.096	0.19	0.12
Clustering coefficient	0.87	0.9	0.8	0.85

A relatively high diversity of the substrate binding proteins in *Devosia* unveiled the high nutritional demands and efficiency of the genus towards uptake of a wide range of structurally and chemically diverse amino acid side chains from environment. Apart from nutritional significance, the permeases are also gates to acquire natural and non-natural cargo molecules attached with amino acid side chains of peptides thereby acting as environmental sensors^37,38^. These signals drive the bacterial chemotaxis and form the basis of bacterial tolerance and bioremediation of environmental pollutants by bacteria^39^. Thus, the genus might as well have adopted this strategy for chemosensing and mediating signals to help them regulate their cellular processes for tolerating environmental stress.

### Genome scale positive selection

For determining the genes under positive selection pressure, the orthologous gene clusters identified in all the genomes were filtered for eliminating clusters with low quality sequences. A total of 2000 valid clusters thus obtained were tested for presence of recombination and filtered based on FDR < 10% and dN/dS values were calculated. The dN/dS values compare the rate of substitutions at non-synonymous sites (dN) with the rate of substitutions at synonymous sites (dS) in protein orthologs. Values greater than 1 indicates positive selection while values less than one indicate that the protein is under purifying selection. The genes which were present in at least 25 genomes (1263 gene clusters) were considered to denote the positively selected genes of the genus. 24 genes were found to be under positive selection pressure with dN/dS values (ω) greater than 1 (Table 3).

**Table 3:** List of genes identified to be under positive selection across the genus.

Gene	Function	ω	p-value	q-value
Pyrroline-5-carboxylate	Proline synthesis and osmotic stress	15.385168	0.000456	0.004044
Alpha/beta hydrolase	Hydrolysis	13.417266	0.00122	0.008221
LamB	Lactam utilization	12.54433	0.001888	0.012231
Response regulator in two-component regulatory system with PhoQ	Response to divalent cation starvation; Resistance to antimicrobial peptides	21.08824	0.000026	0.000738
Translation initiation factor 3	Translation	14.490274	0.000714	0.005226
Acetyl-coenzyme A carboxyl transferase alpha chain	Membrane lipid synthesis	17.42453	0.000165	0.001848
probable iron binding protein from the HesB_IscA_SufA family	Iron starvation	20.783648	0.000031	0.000738
Exopolyphosphatase (EC 3.6.1.11)	Inorganic polyphosphate utilization, adaptation to amino acid starvation	17.073976	0.000196	0.002064
NifU-like domain protein	Maturation of nitrogenase; scaffold for Fe-S cluster assembly	11.071378	0.003943	0.02372
DNA-directed RNA polymerase omega subunit (EC 2.7.7.6)	Transcription	11.501884	0.00318	0.019835
Molybdopterin biosynthesis protein MoeB	Cofactor for detoxifying enzymes	9.045598	0.010859	0.053791
Transcriptional regulator, LuxR family	Quorum sensing, motility	19.678534	0.000053	0.000999
Glutamate methylesterase CheB (EC 3.1.1.61)	Chemotaxis	14.858994	0.000593	0.00476
MutT/nudix family protein	Housekeeping enzyme	10.824536	0.004462	0.025911
hypothetical protein	—	18.851394	0.000081	0.001132
FtsZ (EC 3.4.24.-)	Cell division	33.57748	0	0.000009
SSU ribosomal protein S6p	Ribosomal protein	17.630638	0.000148	0.001786
Scaffold protein for [4Fe-4S] cluster assembly ApbC, MRP-like	Fe-S cluster assembly; Probable Iron binding protein	24.659618	0.000004	0.000227
PetP	HTH-type transcriptional regulator	9.34578	0.009345	0.049185
3-isopropylmalate dehydratase small subunit (EC 4.2.1.33)	Biosynthesis of leucine and lysine	9.057016	0.010797	0.053791
Ribonuclease PH (EC 2.7.7.56)	tRNA processing	18.16992	0.000113	0.001469
Hypothetical protein	—	23.900184	0.000006	0.000227
Universal stress protein UspA and related nucleotide-binding proteins	Response to various stressors	14.555118	0.000691	0.005226
Sulfur acceptor protein SufE for iron-sulfur cluster assembly	Oxidative stress and iron starvation	19.676042	0.000053	0.000999

The genes related to growth, osmotic stress response, inorganic polyphosphate utilization and amino acid and divalent cation starvation were under strong positive selection pressure. Apart from these, the gene responsible for cofactor molybdopterin synthesis was found to be under strong positive selection pressure. Molybdopterin acts as a cofactor for many enzymes responsible for detoxification such as sulphite oxidase, xanthine oxidase, aldehyde oxidase and formate dehydrogenase^40^. These molybdopterin dependent enzymes which were present in the genomes enable the optimal growth of strains by utilization of nitrate, inorganic sulfur and purines and pyrimidines as carbon and nitrogen sources. The genes involved in assembly of iron-sulfur (Fe-S) clusters were under positive selection pressure. Fe-S clusters are cofactors of proteins that perform a number of biological roles including electron transfer, redox and non-redox catalysis, and sensing for iron^41^. Besides, the universal stress protein (UspA) that gets activated in response to various stressors such as high temperature and salinity, antibiotics, nutrient starvation^42^ and LuxR family transcriptional regulator that plays a key role in quorum sensing, motility, and antibiotic synthesis^43^ were also positively selected. These positively selected genes signify the evolving environmental tolerance mechanisms among *Devosia* species.

### Determination of positively evolving genes at HCH contaminated sites and differential osmotic stress response

As the three strains IPL18, IPL20 and L15 isolated from HCH contaminated sites tolerate high levels of the chlorinated pollutant (450 mg/g of soil)^44^, we looked specifically at their genomic repertoire to uncover what enables them withstand high HCH stress. Through delineation of their orthologous proteins, we identified that their tolerance may be attributed to the abundance of two-component systems such as chemosensory *phoB/phoR, cheA/cheW*, *cheB/cheR, cheD, cheY* and methyl accepting chemotaxis protein I, might as well have been adopted to tolerate HCH stress as has been reported previously in a *Pseudomonas* genotypes^27,45^.

**Figure 6:**
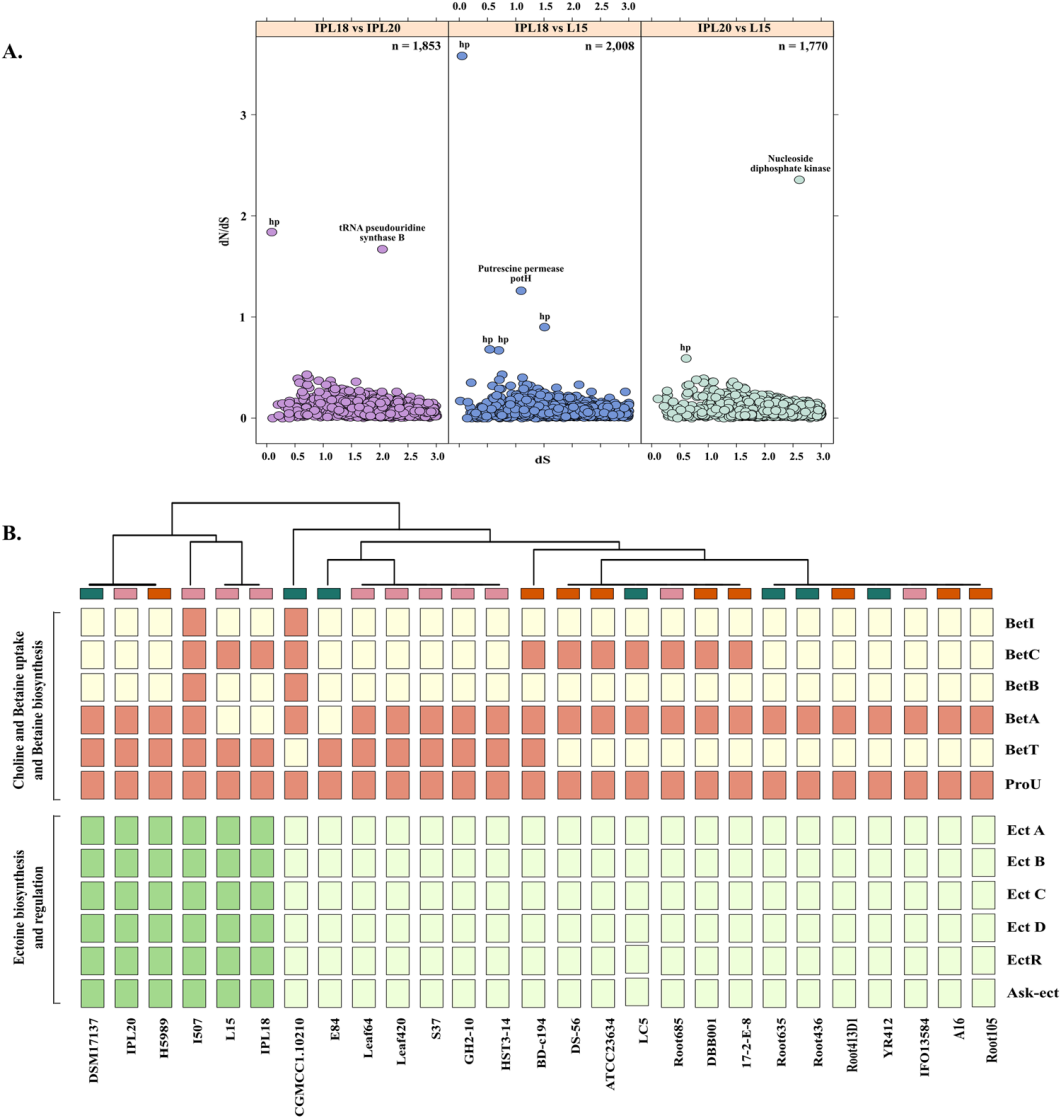
(**A**) Positively selected genes in genome pairs of strains isolated from hexachlorocyclohexane (HCH) contaminated sites. dN/dS values are plotted against dS values. The total number of predicted orthologs are for each pair that were subjected to the analysis are shown. The positively evolving poteins with dN/dS values > 1 are labelled. Hypothetical proteins are denoted as hp. (**B**) Presence absence pattern of the genes involved in the biosynthesis of osmolytes glycine betaine, ectoine and hydroxyectoine in response to osmotic sress response.

In order to determine the proteins encoded within their genomes that are under positive selection pressure to tolerate HCH stress, the orthologous proteins in independent pairs of three strains were subjected to positive selection detection. The majority of the proteins of all pairs were identified to be evolving under purifying selection with dN/dS values < 1 suggesting a conserved repertoire of genes is required for their survival (Fig. 6A). In IPL18 and IPL20, tRNA pseudouridine synthase subunit B was found to be under positive selection pressure (dN/dS = 1.7). Formation of pseudouridine is one of the important post-transcriptional modifications of the tRNAs. Most often these residues are confined to the functionally important part of tRNAs such that the genetic mutants lacking pseudouridine residues exhibit slow growth rates due to difficulties in translation and are not able to compete with wild type cells^46^. Therefore, the enzyme might confer selective advantage during competition at such a challenging niche^47^. In IPL18 and L15, putrescine transporter PotH was positively evolving (dN/dS = 1.25), which transports putrescine and is again involved in growth, as well as incorporation into the cell wall and biosynthesis of siderophore^48^. In IPL20 and L15, nucleoside diphosphate kinase showed dN/dS = 2.3. The enzyme facilitates bacterial cell growth and proliferation and mediates signal transduction^49^. Along with these, many hypothetical proteins were found to be under positive selection pressure (Fig. 6A). The hypothetical protein with the highest dN/dS of 3.58 belonged to GPCR family2-like protein with a query coverage of 76% using SmartBLAST (http://blast.ncbi.nlm.nih.gov/blast/smartblast/). In concordance with the previous results, all the positively selected proteins were related to growth or signalling mechanisms indicating the need to improve genetic fitness to cope high microbial competition at this nutrient depleted site.

As the soils near the dumpsites are also reported to have high salinity levels^44^, we compared the profiles of osmotic stress response of these strains to determine any active gene transfers at this dumpsite and to gain insights on the plasticity of the genus *Devosia*. One of the strategies to cope osmotic stress is the uptake and synthesis of osmolytes such as glycine betaine, ectoine and hydroxyectoine^50^. Glycine betaine is synthesized from choline by betICBA operon where BetI is a sensory repressor and BetC converts choline-O-sulfate into choline. Choline uptake is mediated by BetT or ProU which is converted to glycine betaine by dehydrogenases BetA and BetB^51^.The tendency to synthesize the glycine betaine was restricted to I507 and CGMCC1.10210. However, the isolates from HCH dumpsite encoded complete clusters for synthesis of other two osmolytes ectoine and hydroxyectoine (Fig. 6B). Ectoine is synthesized from phosphorylation of aspartate to β-aspartyl phosphate by aspartokinase (Ask) which is then converted to a semialdehyde derivative. The derivative is successively converted to ectoine by ectABC gene cluster regulated by ectR^52^. Hydroxyectoine is produced from ectoine by a hydroxylase (EctD)^53^. The complete pathway for their synthesis was also determined in DSM17137, H5989 and I507 but was altogether absent in all other strains (Fig. 6B). The isolates from HCH and strain I507 appear to have acquired the potential for synthesis of ectoine and hydroxyectoine to overcome the osmotic stress posed by the high salinity in their respective niches.

### Degradation of organic compounds

#### Utilization of phosphonates and sulphonates

The sulphonates and phosphonates are added to environment through pesticides and are major source of sulfur and phosphorus in the soils^54,55^. Bacterial degradation of organic P and S play large role in global P and S cycling. As the *Devosia* are optimized for efficient utilization of nutrients, it evoked our interest in genus wide profiles for degradation of organic P and S.

**Figure 7:**
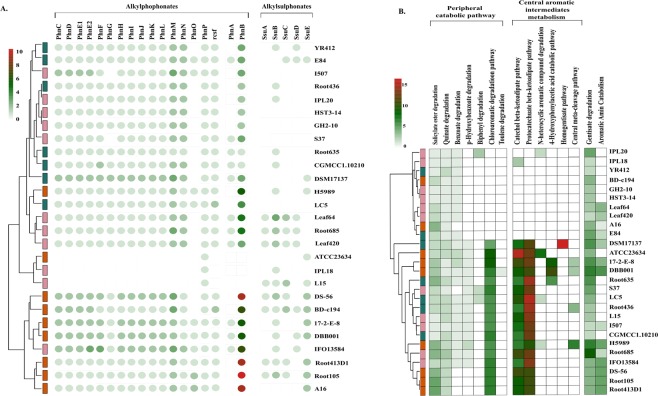
Biodegradation of organic compounds. Clustering of genomes based on the ability to degrade (**A**) alkylphosphonates and alkanesulphonates and (**B**) aromatic and xenobiotic compounds. The genomes are colored according to their original phylogenetic clustering at the tip of each branch in the tree.

Bacterial degradation of complex C-P bond in alkylphosphonates is catalyzed by C-P lyase encoded by a 14 gene cluster *phnCDEFGHIJKLMNOP* in which *phnGHIJKLM* code for the “core” components of the enzyme, PhnJ catalyzes the central reaction while *phnNOP* gene products play accessory roles^56,57^. *phnCDE* encode an ABC transporter and *phnF* a repressor protein. *rcsF* encodes a phosphoesterase analogous to phnP^58^. The degradation of aliphatic sulfonates is mediated by *ssuEADCB* gene cluster where SsuABC proteins constitute an ABC transport system while SsuD catalyzes the desulfonation of substrates and SsuE is an FMN reductase^59^. Our analysis revealed that the degradation of alkylphosphonates was widespread across *Devosia* while differential profiles for the degradation of alkylphosphonates were observed among the strains (Fig. 7A). Strains ATCC23634, IPL18 and L15 completely lacked potential to degrade alkylphosphonates (Fig. 7A). We argue that the strains IPL18 and L15 might have lost the catabolic ability in the process to tolerate the dominant pollutant *i.e*., HCH in their habitats. These functions are presumed to have been of environmental origin based on the clustering of genomes independent of their phylogeny. Overall, the analysis highlighted plasticity of *Devosia* genomes with potential for continued influx of novel functions and their evolution in response to environment.

### Degradation of aromatic and xenobiotic compounds

The degradation of aromatic compounds by bacteria has immense environmental significance as they are the most prevalent class of natural carbon compounds which are also persistent pollutants^60^. So far, genera such as *Pseudomonas, Acinetobacter, Geobacter, Dechloromonas* and *Novosphingobium* have been extensively studied for their abilities of aromatic compounds degradation^61–66^. In this study, we examined the enzyme arsenal for remediation of aromatic compounds encoded wide the genus *Devosia*. The genomes were rich in the genes involved in both the branches of ß-ketoadipate utilization, one that converts catechol derived from various aromatic hydrocarbons, amino aromatics, and lignin monomers to beta-ketoadipate and another that converts protocatechuate, derived from phenolic compounds also to beta-ketoadipate for reduction through tricarboxylic acid cycle^67^. Among the peripheral catabolic pathways, the degradation of chloroaromatic compounds was most abundant among the strains (Fig. 7B). Again, the strains did not cluster in concordance with their phylogenetic distances. To note, strain DSM 17137 which showed maximum divergence with respect to overall functional profiles displayed maximum potential for homogentisate degradation pathway which were lacked by all other strains further confirming its functional divergence. In line with the previous observations, strain ATCC23634, the freshwater isolate was again the next most diverged among all analyzed genomes which displayed maximum potential for degradation of heterocyclic aromatic compounds (Fig. 7B). Overall, the profiles led us to consider that *Devosia* have acquired the potential of bioremediation during the course of evolution to adapt optimally to the environmental insults imposed on them. The conclusion was supported by the fact that the strains did not cluster based on their phylogeny but rather based on their abilities to degrade wide array of aromatic and xenobiotic compounds such as benzoate, p-hydroxybenzoate, biphenyl, catechol and chlorinated aromatic compounds.

### Metabolic versatility for decomposition of urea

**Figure 8:**
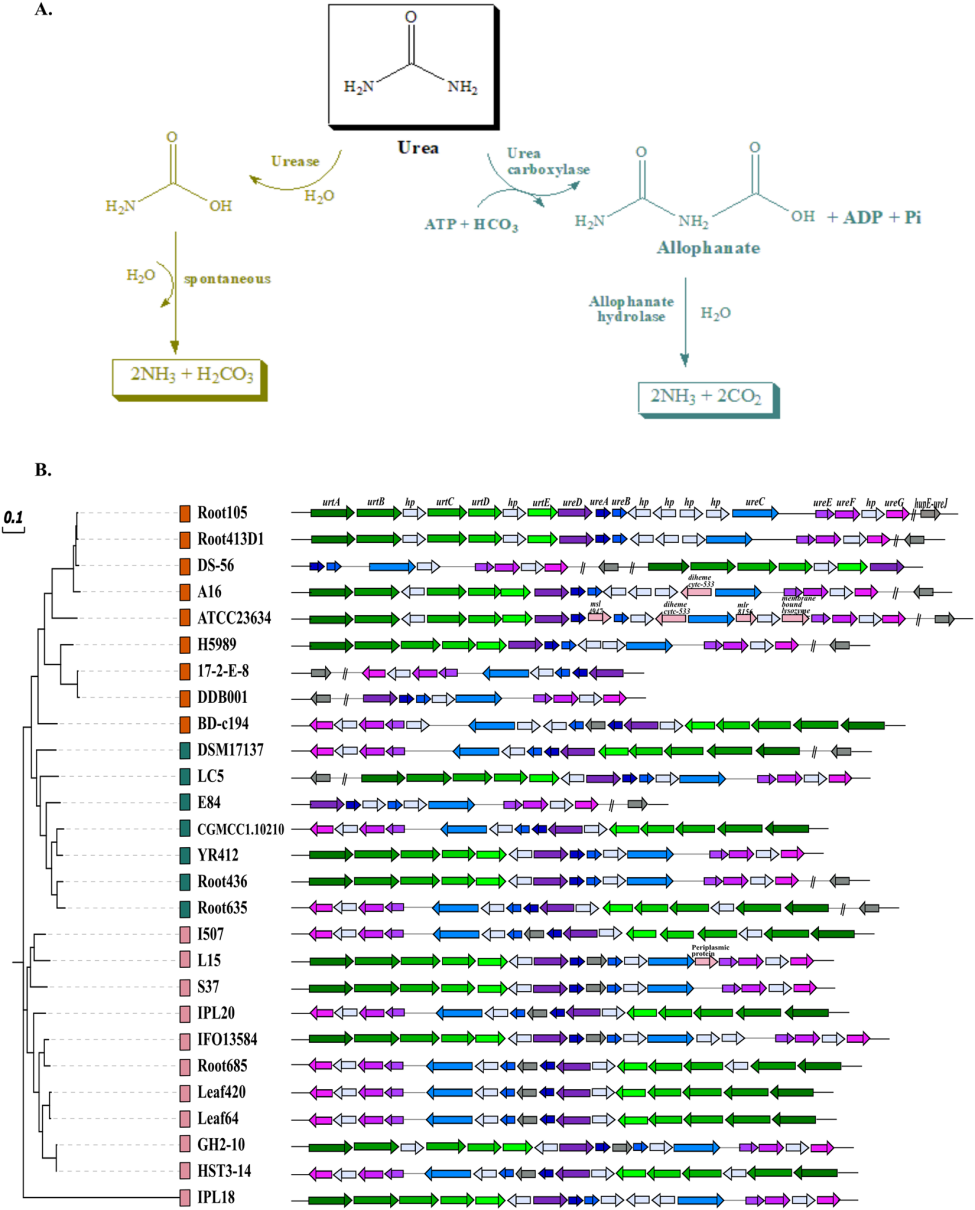
Metabolic versatility of urea decomposition. (**A**) The two different metabolic routes of decomposition of urea catalyzed by different enzymes namely urease and urea carboxylase. (**B**) A phylogram based on the genes involved in the urease pathway and their organization into operons within genomes. The phylogenetic clades are shown with the colored boxes in front of each genome name in the tree.

Urea occurs as a source of organic nitrogen and its decomposition by bacteria is of immense significance for bacterial growth and nutrient cycling. Urea may be decomposed by either of the two different enzymatic pathways catalyzed by urease and urea amidolyase as illustrated in Fig. 8A ^68^. The second pathway catalyzed by urea amidolyase comprises activities of urea carboxylase and allophanate hydrolase^69^. This alternative pathway was only detected in few genomes (data not shown) and therefore, was not further inspected. Urease pathway was found to be the core pathway for urea decomposition as all the essential genes *ureA, ureB, ureC* encoding a functional urease and several accessory protein encoding genes *ureDEFG*, *ureI* or *ureJ*^70^ were present in all genomes (Fig. 8B). However, the genes for uptake of urea, *urtABCDE* were absent in DDB001, 17-2-E-8 and E84 that might have lost them or that might also harbor unique genes that still need to be characterized. Notably, the *ureC* gene coding for the α-subunit of urease was found to be evolving in strain DS-56 under strong positive selection pressure (dN/dS = 3.19). The *ureC* is the largest of the genes encoding urease functional subunits and is essential for a functional urease^70,71^. The strain DS-56 was isolated from the island soil near sea where urea acts as the dominant N source and thus the organism might be dependent upon its decomposition for building amino acids and hence proteins. We further tried to reconstruct the phylogeny in order to check the conservedness of the genes belonging to this pathway. The maximum-likelihood phylogeny was similar to phylogeny based upon conserved genes and marker proteins. This suggests that urea decomposition by urease is a conserved function of the genus. The conserved organization of the genes within operons also provided evidence of phylogenetic origin of this pathway.

### Determination of toxin-antitoxin (TA) systems

Bacterial toxin-antitoxin (TA) systems are key regulators of cellular processes that can respond to external stimuli and promote survival during periods of stress^72^. A TA locus is composed of two genes coding for a toxin and its cognate antitoxin^73^. Under favourable conditions, antitoxins typically inhibit their cognate toxins. While they are readily proteolysed upon stress encounters thereby unleashing the inhibitory effect of the toxin^72^. Widespread TA loci could be dissected within *Devosia* that all belonged to type II class in which both the toxin and anti-toxin are proteins^73^. Among the major TA systems within the genus were *higB/higA* and *vapC/vapB* but others such as *parE/parD, yoeB/yefM, yafQ/dinJ* and *relB/relE* were also present (Table 4). These small genetic modules are thought to epigenetically regulate bacterial survival controlling a wide range of biological functions including growth, persistence, programmed cell death, phage inhibition, biofilm formation and response to stress^72,74^. Besides, these loci are also known to stabilize the mobile genetic elements (MGEs) and enhance the genomic plasticity^72^. Therefore, the study could present a scenario that the environmental stress could have favored the accumulation of TA systems that confer selective advantage and competitiveness to the genus.

**Table 4:** Various toxin-antitoxin (TA) systems identified within *Devosia* genomes.

Genomes	Toxins and Antitoxins												
	RelB/StbD	RelE/StbE	ParE	ParD	HigB	HigA	VapC	VapB	VapB1	YoeB	YefM	YafQ	DinJ
DDB001	0	0	0	0	1	1	1	0	0	0	1	0	0
17-2-E-8	0	0	0	0	1	1	0	0	0	1	1	0	0
L15	0	0	0	0	1	1	0	0	0	1	0	0	0
GH2-10	0	0	0	1	1	1	0	0	0	0	0	0	0
HST3-14	0	0	0	1	1	1	0	0	0	0	0	0	0
S37	0	0	0	1	0	0	1	1	0	0	0	0	0
DSM17137	0	0	0	0	0	0	1	1	0	0	0	0	0
Root685	0	0	0	0	1	1	2	0	0	0	0	0	0
IPL20	0	0	0	0	1	1	1	0	0	0	0	0	0
BD-c194	0	0	1	1	3	2	4	5	1	1	1	0	0
Root635	0	0	0	1	2	2	1	0	0	0	0	1	1
E84	0	0	0	0	0	0	1	1	0	0	0	0	0
A16	0	0	1	1	0	3	0	1	0	0	0	0	0
Root105	0	0	1	1	1	1	2	2	0	0	0	0	1
CGMCC 1.10210	0	0	0	1	1	2	2	2	0	0	1	0	0
IFO13584	0	0	0	0	1	1	2	2	0	0	0	0	0
LC5	0	0	1	2	0	1	1	1	0	0	0	0	1
YR412	0	1	0	1	1	1	1	1	0	1	1	0	0
Root413-D1	0	0	1	1	1	1	2	2	0	0	0	0	1
H5989	0	0	1	1	0	1	0	0	0	0	0	0	0
Leaf420	0	0	0	2	0	0	0	0	0	0	0	0	0
ATCC 23634	1	0	0	2	0	1	2	2	0	0	0	0	1
Leaf64	0	0	0	1	0	0	0	0	0	0	0	1	1
Root436	0	0	0	1	2	2	1	0	0	0	0	1	1
DS-56	1	1	3	2	0	0	5	2	0	1	1	1	0

## Conclusions

In the present study, the genomes of 27 strains of the genus *Devosia* were analyzed which allowed the description of the open pangenome of the genus with half of the pangenome (50.32%) represented by the unique genes suggesting the role of their respective environments in shaping the genomic repertoire of the members. This was also indicated from the dissimilar phylogenetic pattern obtained based on conserved core genes and those obtained from the reconstruction of overall metabolic profiles. The phylogenetic relationships of the strains could be clearly resolved by the study. The clustering of the strains based on specific bioremediation linked functions and niche specific adaptations for example, the synthesis of osmolytes, utilization of sulphonates and phosphonates and degradation of aromatic and xenobiotic compounds revealed their plastic genomic repertoire subject to locally relevant environmental stressors. The uptake and utilization of nutrients for growth and survival was found to be the dominant function of the genus along with detoxification and degradation of organic pollutants. On this account, the genes associated with growth, motility, detoxification and nutrient starvation were found to be positively evolving. In concordance, the abundance of ABC class transporters for uptake of di- and oligo-peptides and potential of urea decomposition further revealed that the members have well adapted themselves for survival at hydrocarbons and organic compounds rich habitats by optimizing their genetic repertoire for optimal nutrient uptake and metabolism.

## Materials and Methods

### Genomic DNA extraction and sequencing

*D. crocina* IPL20 and *D. lucknowensis* L15 were isolated from soils contaminated with hexachlorocyclohexane (HCH) from dumpsites located at Chinhat and Ummari villages in Lucknow, India^8,19^. The strains were grown on Luria-Bertani (LB) agar incubated at 28 °C and genomic DNA was isolated by lysis with lysozyme and proteinase K followed by CTAB extraction using method described elsewhere^75^. Sequencing was performed on an Illumina HiSeq. 2500-1TB platform with Illumina regular fragment library of insert size 300 bp. A paired end library of read length 151 bp was generated for each genome. The sequencing and assembly was performed under the project ‘Genomic Encyclopedia of Type Strains, Phase III’ by the Joint Genome Institute (JGI) [Project ID: 1102317 (*D. crocina* IPL20) and 1102429 (*D. lucknowensis* L15)]. Whole genome sequences are available on NCBI under the accession numbers NZ_FPCK00000000.1 and NZ_FXWK00000000.1 respectively.

### Selection and annotation of genomes

The whole genome sequences of all publicly available draft and complete genomes were retrieved from NCBI and JGI databases in March 2018 (n = 33). For all genomes, open reading frames (ORFs) were predicted using Prodigal^76^ and percentage completeness were estimated using 107 essential genes^77^ based on hidden Markov models (HMMs). Using the completeness criterion, we selected 27 strains (>96% complete) for comparative analyses (Table 1). Further, the putative protein-encoding genes were also predicted using GLIMMER-3^78^ on RAST server v2.0^79^. The rRNAs and tRNAs were predicted using RNAmmer v1.2^80^ and ARAGORN^81^, respectively. The clustered regularly interspaced short palindromic repeat (CRISPR) elements were identified using CRISPR Finder^82^. Phage and prophage regions were determined using PHASTER^83^.

### Phylogenomics analysis

The maximum likelihood phylogeny based on 400 ubiquitous and conserved marker proteins, was constructed using PhyloPhlan^30^ with 1000 bootstrap replications. iTOL v3 was used to visualize the tree^84^. In addition, phylogenetic analysis was also performed on the core genes identified in single copy within each genome. For this, amino acid alignments for each gene cluster were generated using KAlign v2.04 that employs Wu-Manber string-matching algorithm, to improve the accuracy of multiple sequence alignment^85^. The concatenated alignments were used to construct a maximum likelihood tree based on LG + F + R6 identified as the best fit model in IQ tree v1.6^86^. The model generates a general amino acid replacement matrix^87^ using empirical amino acid frequencies and FreeRate model for calculating heterogeneity across sites. For genome-wide reconstruction of phylogeny, blast based pairwise Average Nucleotide Identity (ANIb) values computed using JSpecies web server^88^ were used to construct a Pearson correlation matrix and plotted in R (R Development Core Team, 2015).

### Pan-gene clusters and identification of homologues

The pan-gene clusters were identified using microbial pangenomics workflow in anvi’o^33^ and the genomes were organized based on the distribution of gene clusters using MCL algorithm into core, dispensable and strain-specific contents (Distance: Euclidean; Linkage: Ward). The genes were annotated by BLASTp against the NCBI COG database. Heatmap based on the annotated COG functions of the core and singleton gene clusters were then plotted in R (R Development Core Team, 2015). The Tettelin best-fit curves^32^ of core and pangenomes were constructed using OMCL v1.4 implemented in GET_HOMOLOGUES pipeline^89^.

### Comparative functional analyses

Functional annotation of genes was done on RAST v2.0^69^ using the SEED subsystems approach. The ORFs were annotated by KAAS (KEGG Automatic Annotation Server)^90^ using Bi-directional Best Hit (BBH) algorithm. The top 50 metabolic pathways reconstructed within each genome using MinPath^91^ were plotted as heatmap using pheatmap package^92^ in R (R Development Core Team, 2015).

### Sequence similarity network analysis

The di- and oligo-peptide permeases were identified within the genomes using Protein BLAST on NCBI database. The sequences were analysed by constructing similarity networks in which the relationships were read as independent pairwise alignments. The approach offers serious advantages over the phylogenetic trees in inferring relationships between large sequence data sets at defined cut-offs with ease. The sequences were filtered for the removal of 100% identical sequences using CD-HIT^93^. A pairwise BLAST of all non-redundant proteins was performed and sequence similarity networks (SSN) were constructed with a threshold alignment score of 50%. The threshold cutoff values of 1e-30 and 1e-25 were used for construction of opp and dpp sequence networks respectively upon analysing the trends of varying alignment length at different e-values. The networks were visualized in Cytoscape v3.6.1. The average numbers of neighbors or degree for a node or sequence was calculated as:

$$k=\frac{{2}K}{N}$$

where *K* denotes the total number of edges and *N* denotes the total nodes. To estimate the diversity/similarity among sequences, the density of networks i.e. the fraction of all edges in the similarity networks was also calculated as:

$$D=\frac{{2}K}{N(N-{1})}$$

### Genome scale and pairwise positive selection detection

The orthologous gene clusters were determined using OrthoMCL v1.4. Orthologous groups with single copy genes were then filtered for determining orthologs under positive selection using POTION v1.1.3^94^. Groups with evidence of recombination were removed from analysis using PhiPack^95^ that integrates three recombination tests: Phi, NSS and Max Chi2. For each group, multiple protein sequence alignments were generated using MUSCLE v 3.8.31 and trimmed using TrimAl v1.2^96^. DNAML from phylip was used for phylogenetic tree reconstruction with 100 bootstraps. Later, groups were tested for positive selection using site-model analysis in codeml and a likelihood ratio test was conducted. The *p*-values were calculated as 2Δℓ (twice the difference in likelihood of the two nested models evaluated) based on the *χ*^2^ distribution with 2 ° of freedom followed by multiple hypothesis correction. Errors were minimised through False Discovery Rate (FDR) adjusted q-values (significance threshold cutoff of 10%).

To determine the evolutionary pressures at the HCH dumpsites, dN/dS values were calculated independently for the three HCH tolerating strains in a pairwise manner. The orthologous proteins were aligned using KAlign v2.04 and further converted to corresponding codon alignments using PAL2NAL script^97^. yn00 module in the PAML package was used to calculate dN/dS value for each orthologous pair.

## Supplementary information

Supplementary information.
