# Supplementary material for: Defining the Environmental Adaptations of Genus Devosia: Insights into its Expansive Short Peptide Transport System and Positively Selected Genes
Source: Sci Rep. 2020 Jan 24;10:1151. doi: 10.1038/s41598-020-58163-8 (PMC6981132; doi:10.1038/s41598-020-58163-8)
Supplement: Supplementary file 1 — Supplementary information. [file 41598_2020_58163_MOESM1_ESM.pdf]

Supplementary Information

Defining the Environmental Adaptations of Genus *Devosia:* Insights into its Expansive Short Peptide Transport System and Positively Selected Genes

Chandni Talwar[a†], Shekhar Nagar[a†], Roshan Kumar[b], Joy Scaria[c,d], Rup Lal[e] and Ram Krishan Negi[a*].

[a]Department of Zoology, University of Delhi, Delhi-110007, India.
[b]P.G. Department of Zoology, Magadh University, Bodh-Gaya-824234, Bihar, India
[c]Department of Veterinary and Biomedical Sciences, South Dakota State University, Brookings, SD, USA.
[d]South Dakota Center for Biologics Research and Commercialization, SD, USA.
[e]NASI Senior Scientist Platinum Jubilee Fellow, The Energy and Resources Institute, Darbari Seth Block, IHC Complex, Lodhi Road, New Delhi-110003, India

† Authors contributed equally

Email: negigurukul@gmail.com

Figure S1

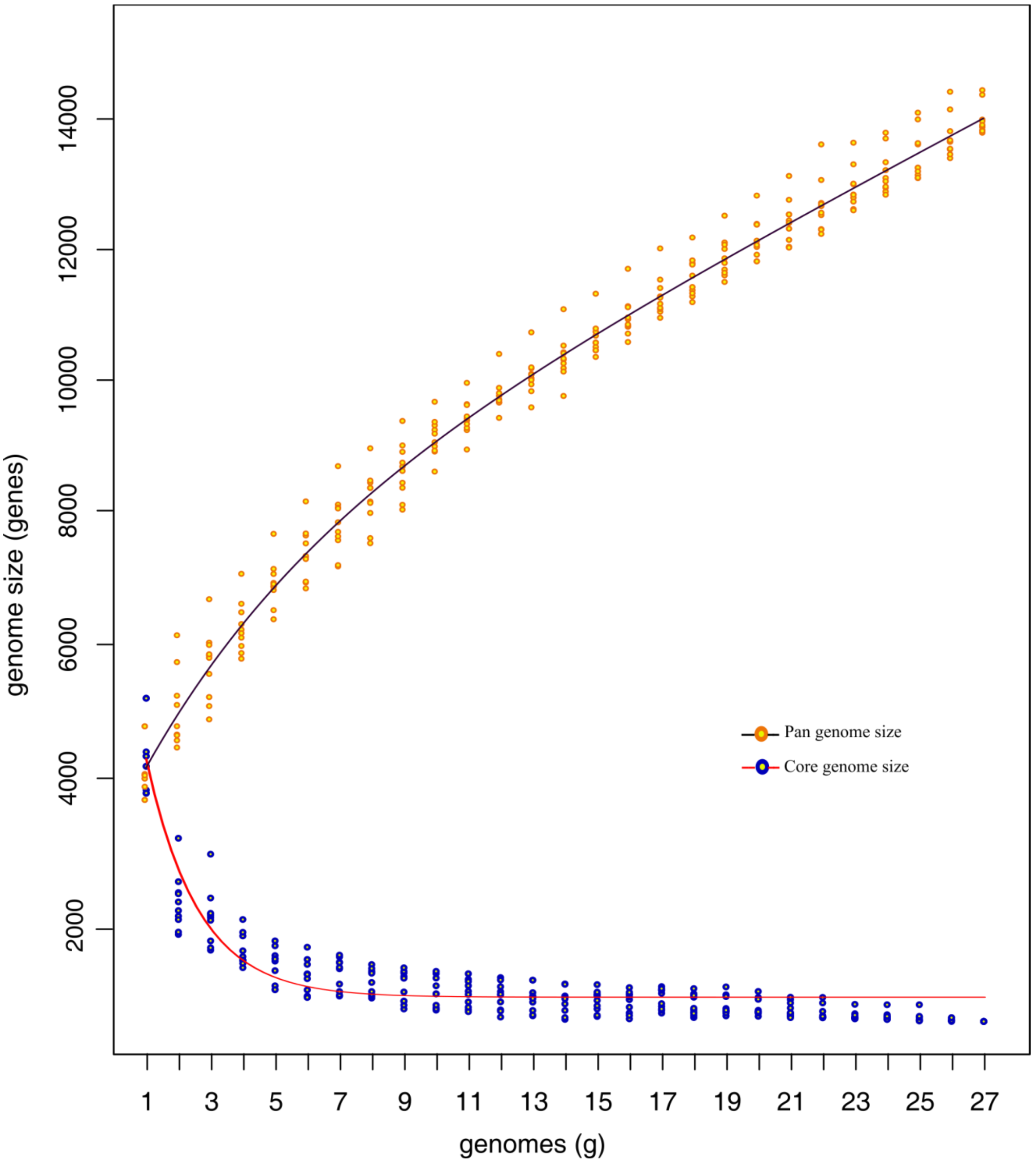

Supplementary Figure S1. Pan and core genome sizes. The numbers of pan and core genes are plotted as a function of the number of genomes (g) added sequentially. y-*axis* shows the change in core genome and pan genome sizes as a function of random addition of genomes plotted on x-*axis*. The sizes of core and pan genomes were computed using OMCL algorithm. The best fit regression represents the Tettelin curve for both core and pan genomes.

Figure S2

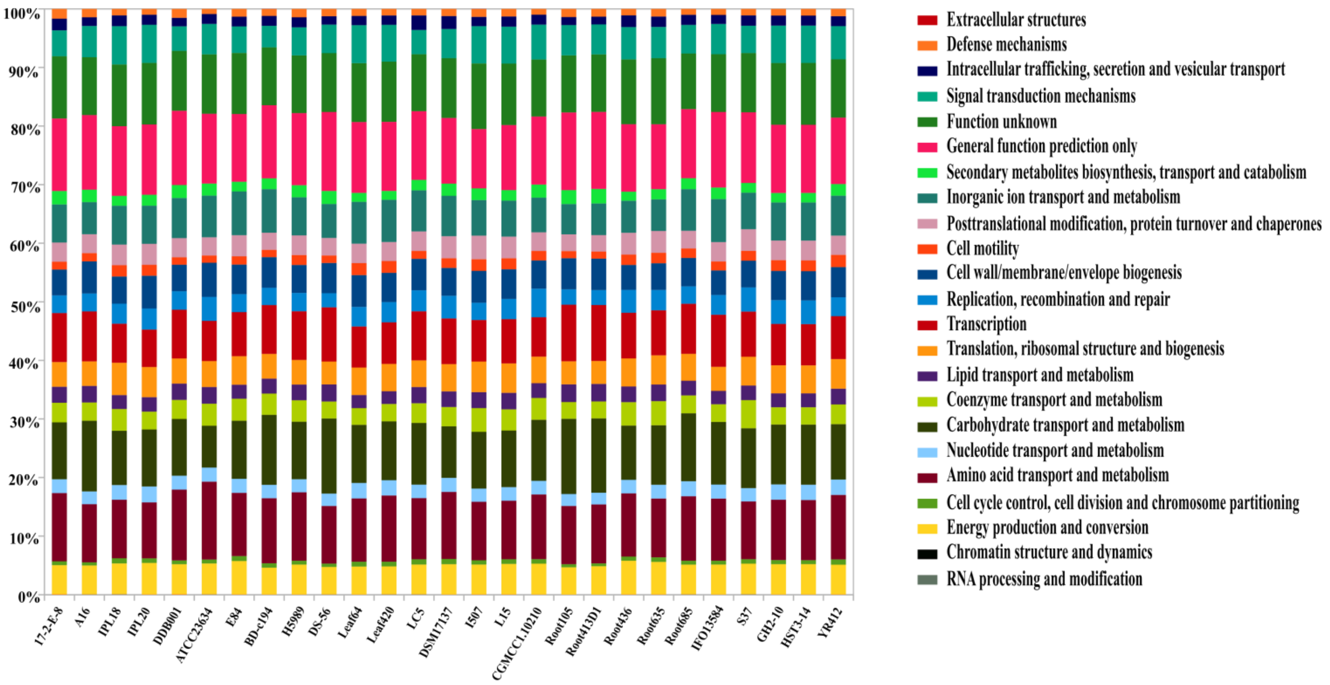

Supplementary Figure S2. Predicted oligo-peptide and di-peptide permeases in the analyzed strains.

Figure S3

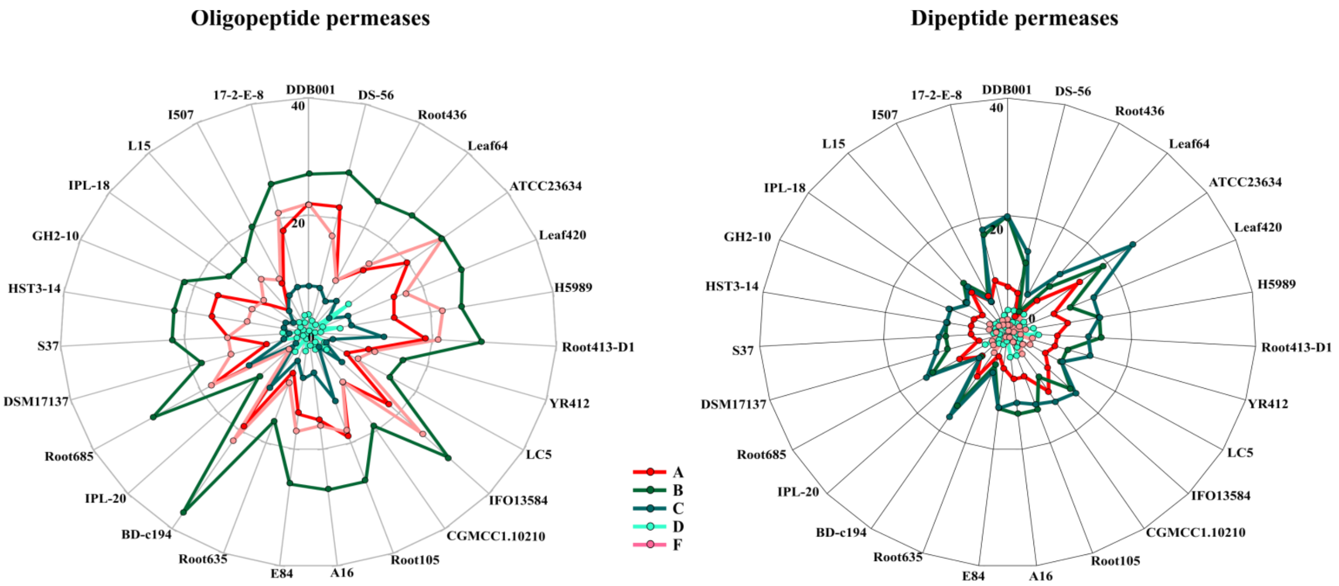

Supplementary Figure S3. Phylogeny of the predicted substrate binding components of (A) oligo-peptide permeases (OppA) and (B) di-peptide permease (DppA) proteins predicted in the genomes of *Devosia* (blue) and representative genomes of other genera of family *Hyphomicrobiaceae* (red) constructed using Neighbour-joining method at 100 bootstrap replications. The boostrap confidence levels are shown with circles on the branches.

Supplementary Table S1. List of genomes of the family *Hyphomicrobiceae* with the predicted copy number of genes associated with oligo-peptide and di-peptide transporters included in the study for comparison with *Devosia*.

**Genome**	**OppA**	**OppB**	**OppC**	**OppD**	**OppF**	**DppA**	**DppB**	**DppC**	**DppD**	**DppF**
Aquabacter sp. Sn-9-2 (NZ_RWKV00000000.1)	2	3	2	2	2	3	2	2	0	0
Aquabacter spiritensis DSM 9035 (NZ_SMAI00000000.1)	2	3	1	2	2	2	2	2	0	0
Arsenicitalea aurantiaca 42-50 (NZ_RZNJ00000000.1)	8	14	3	3	9	6	10	13	2	1
Blastochloris sp. GI (NZ_AP018907.1)	1	2	1	0	0	0	0	0	0	0
Blastochloris viridis ATCC 19567 (NZ_CP012946.1)	1	2	1	0	0	0	0	0	1	0
Cucumibacter marinus DSM 18995 (NZ_AUCR00000000.1)	6	12	4	2	11	5	8	7	2	1
Dichotomicrobium thermohalophilum DSM 5002 (NZ_QXDF00000000.1)	1	2	1	0	0	0	0	0	0	0
Filomicrobium insigne CGMCC1.6497 (NZ_FNJC00000000.1)	1	2	1	0	0	0	0	0	0	0
Filomicrobium sp. MED665 (NZGY00000000.1)	4	6	1	0	5	2	1	6	0	0
Hyphomicrobium denitrificans ATCC 51888 (NC_014313.1)	1	2	1	0	0	0	0	0	0	0
Hyphomicrobium facile DSM 1565 (NZ_FPCH00000000.1)	1	2	1	0	0	0	0	0	0	0
Hyphomicrobium sp. MC1 (NC_015717.1)	1	2	1	0	0	2	0	0	0	0
Hyphomicrobium sulfonivorans WDL6 (NZ_LMTR00000000.1)	1	4	1	0	0	1	0	1	0	0
Hyphomicrobium zavarzinii ZV622 (NZ_ARTG00000000.1)	1	2	1	0	0	0	0	0	0	0
Maritalea mobilis CGMCC 1.7002 (NZ_SNYR00000000.1)	6	10	4	5	9	8	10	9	1	3
Maritalea myrionectae DSM 19524 (AUHV01)	3	7	3	3	5	8	6	7	1	3
Pelagibacterium halotolerans B2 (NC_016078.1)	5	17	3	0	12	9	9	10	1	0

Pelagibacterium lentulum B2 (NZ_RZMW00000000.1)	8	15	3	5	12	9	15	12	2	2
Pelagibacterium luteolum CGMCC 1.10267 (NZ_FNCS00000000.1)	3	11	3	2	8	6	5	6	1	0
Pelagibacterium montanilacus CCL18 (NZ_RZMX00000000.1)	1	8	3	5	7	6	3	5	0	0
Prosthecomicrobium hirschii 16 (LJYW01)	8	12	2	2	16	12	15	18	2	0
Prosthecomicrobium sp. SZUA402 (QKBQ00000000.1)	1	1	1	1	0	4	2	1	0	1
Rhodomicrobium udaipurense JA643 (NZ_JFZJ00000000.1)	1	2	1	0	0	0	0	0	0	0
Rhodomicrobium vannielii ATCC 17100 (NC_014664.1)	1	2	1	0	0	0	0	0	0	0
Rhodoplanes elegans DSM 11907 (NZ_NPEU00000000.1)	1	4	1	0	2	4	2	2	0	0
Rhodoplanes piscinae DSM 19946 (NPEW01)	1	2	1	0	0	0	0	0	0	0
Rhodoplanes roseus DSM 5909 (NZ_NPEX00000000.1)	1	4	1	0	2	5	2	2	0	0
